# A DNA Based Biosensor Amplified With ZIF-8/Ionic Liquid Composite for Determination of Mitoxantrone Anticancer Drug: An Experimental/Docking Investigation

**DOI:** 10.3389/fchem.2020.00814

**Published:** 2020-10-20

**Authors:** Marzieh Alizadeh, Parviz Aberoomand Azar, Sayed Ahmad Mozaffari, Hassan Karimi-Maleh, Ali-Mohammad Tamaddon

**Affiliations:** ^1^Department of Chemistry, Science and Research Branch, Islamic Azad University, Tehran, Iran; ^2^Department of Chemical Technologies, Iranian Research Organization for Science and Technology (IROST), Tehran, Iran; ^3^Laboratory of Nanotechnology, Department of Chemical Engineering, Quchan University of Technology, Quchan, Iran; ^4^Center for Nanotechnology in Drug Delivery, School of Pharmacy, Shiraz University of Medical Sciences, Shiraz, Iran

**Keywords:** mitoxantrone, ZIF-8, 1-butyl-3-methylimidazolium methanesulfonate, modified electrode, ds-DNA biosensor, drug analysis

## Abstract

An ultrasensitive DNA electrochemical biosensor based on the carbon paste electrode (CPE) amplified with ZIF-8 and 1-butyl-3-methylimidazolium methanesulfonate (BMIMS) was fabricated in this research. The DNA/BMIMS/ZIF-8/CPE was used for the selective determination of a mitoxantrone anticancer drug in aqueous solution, resulting in a good catalytic effect and a powerful ability for determining mitoxantrone. Also, the interaction of the mitoxantrone anticancer drug with guanine bases of ds-DNA was used as a powerful strategy in the suggested biosensor, which was confirmed with docking investigation. Docking study of mitoxantrone into the ds-DNA sequence showed the intercalative binding mode of mitoxantrone into the nitrogenous-based pairs of ds-DNA. The effective factors such as ds-DNA concentration, temperature, buffer types, and incubation time were also optimized for the fabricated mitoxantrone biosensor. The results showed that, under optimum conditions (T = 25°C; incubation time=12 min; pH= 4.8 acetate buffer solution and [DNA] = 50 mg/L), the DNA/BMIMS/ZIF-8/CPE could be used in mitoxantrone assay in a concentration ranging from 8.0 nM to 110 μM with a detection limit of 3.0 nM. In addition, recovery data between 99.18 and 102.08% were obtained for the determination of mitoxantrone in the injection samples using DNA/ZIF-8/BMIMF/CPE as powerful biosensors.

## Introduction

Mitoxantrone (developed in the 1980's) is one of the famous anthracycline anti-cancer agents with a wide range of applications in the treatment of breast cancer, acute myelogenous leukemia, and Non-Hodgkin's lymphoma (Lenk et al., 1987; Vollmer et al., 2010). This drug stays in the body for a long time (elimination *t*1/2 = 75 h) and has various side effects such as low blood counts, nausea, vomiting, weakness, low blood pressure, and hair loss (Scott and Figgitt, 2004). Moreover, the highest concentrations of mitoxantrone were detected in the heart, liver, and thyroid (Fox, 2004). Assay of anticancer drugs in biological samples such as blood is one of the most important strategies available to monitor the harmful effects of such drugs on the body (Bolanowska et al., 1983; Baghayeri et al., 2014, 2017, 2018a; Beitollahi et al., 2014; Veisi et al., 2015; Fouladgar, 2018). From the methods reported for measuring the pharmaceutical and biological compounds, electrochemical methods with more advantages such as simplicity of analysis method, low cost, and fast analysis are considered to be more important compared to the other methods (Yuan et al., 2013, 2017; Mozaffari et al., 2014; Eren et al., 2015; Movaghgharnezhad and Mirabi, 2019). Intercalative binding between DNA and mitoxantrone has been proven by the Li group (Li et al., 2005). Accordingly, this makes it possible to design the DNA-based electrochemical biosensors for selective analysis of the drug (Tiwari and Sharma, 2020).

Moreover, the DNA-based biosensors have been reported as powerful tools with a high selectivity for the analytical determination of many compounds, especially for anticancer drugs (Brett et al., 1998; Gooding, 2002; Ozsoz et al., 2003; Ensafi et al., 2011). In this regard, the specific interaction of anticancer drugs with adenine and guanine bases in the complex structure of DNA has been used as an appropriate analytical factor to design new biosensors in the analysis of anticancer drugs (Karimi-Maleh et al., 2018; Khodadadi et al., 2019; Yin et al., 2019). In addition, the sensitivity of the DNA-based biosensors is very low at surface of bare electrodes due to the presence of low-conductivity ds-DNA on the sensor surface (Li et al., 2017, 2020). Correspondingly, this point was introduced as one of the most important problems caused by the conventional DNA-based biosensors (Karimi-Maleh et al., 2020c). To overcome this problem, the DNA-based biosensors are typically amplified using the high-conductivity modifiers such as conductor polymers, organic and inorganic compounds, ionic liquids, and nanomaterials (Cheraghi et al., 2017; Sanati and Faridbod, 2017; Baghayeri et al., 2019; Faridbod and Sanati, 2019).

Nanomaterials such as nanoparticles, nanotubes, and nano porous compounds showed many advantages in different fields and also created a new approach to science (Rahmanian et al., 2015; Xu et al., 2018; Yuan et al., 2019, 2020; Karimi-Maleh et al., 2020a). Accordingly, Nano porous materials like zeolitic imidazolate frameworks (ZIF) are a new type of nanomaterials with a high surface area and metal ion in center and imidazolate linkers (Quang Khieu et al., 2018). Recently, many researchers focused on the usage of ZIF and especially ZIF-8 for the electrochemical applications (Wang et al., 2015). ZIF-8 is very stable in water and other aqueous solutions and could be used as mediator for the fabrication of electrochemical sensors for analysis of electroactive compound in water solution (Banerjee et al., 2008). Although the high surface area of ZIF compounds makes creating a more active surface area for electrochemical sensors possible, its low electrical conductivity is one of the most important problems of its high use in electrochemical sensors (Lu et al., 2020). Therefore, to eliminate this problem, the simultaneous usage of these materials with compounds that have a high electrical conductivity such as conductive polymers and ionic liquids, is recommended (Baghayeri et al., 2018b; Jin et al., 2018; Chen et al., 2019).

Ionic liquids are highly conductive and are a green type of organic compound with a wide range application in different scientific fields (Marr and Marr, 2016; Osada et al., 2016; Atta et al., 2019; Tahernejad-Javazmi et al., 2019; Arabali et al., 2020). Moreover, due to the high conductivity and wide electrochemical range windows, ionic liquids were used as amplifiers with a high quality in the fabrication of electrochemical sensors (Bijad et al., 2013; Beytur et al., 2018; Li et al., 2019; Hojjati-Najafabadi et al., 2020). There are many published scientific papers for the application of ionic liquid coupled with other nanomaterials to create a high quality electrochemical sensor in the environmental and biological compounds analyses (Karimi-Maleh et al., 2020b).

Based on the scientific information reported in previous studies, this study developed a high-sensitivity electrochemical biosensor in terms of the use of DNA as a recognition element for analyzing the mitoxantrone anti-cancer drug. To improve the sensitivity of the DNA-based biosensor, the electrode surface was amplified with ZIF-8 and BMIMF as the conductive modifiers with a high surface. The results showed a good selectivity for the analysis of mitoxantrone anti-cancer drug in drugs samples. The docking investigation confirmed the intercalation interaction between the guanine base and mitoxantrone anti-cancer drugs.

## Experimental

### Instrument and Materials

Electrochemical investigation was performed by electrochemical workstation model Ivium-Vertex connected to an electrochemical Cell (Azar electrode Company, Iran). Moreover, the I-V signals were displayed based on the Ag/AgCl/KCl_sat_ reference electrode's potential. Mitoxantrone hydrochloride, ZIF-8, BMIMF, and DNA (Calf Thymus) were purchased from Sigma-Aldrich. Also, carbon powder and paraffin oil were obtained from Merck Company. In addition, phosphoric acid, boric acid, acetic acid, and Tris hydrochloride were purchased from Across Company. Notably, the stock solution of mitoxantrone hydrochloride (0.001 M) was prepared by dissolving 0.517 g mitoxantrone hydrochloride in 100 mL distillated water under the stirring conditions.

### Preparation of BMIMS/ZIF-8/CPE

The BMIMS/ZIF-8/CPE was prepared by mixing ZIF-8 with carbon powder as the powder components in the ratio 5:95 (w/w), and paraffin oil and BMIMS as the liquid binders in the ratio 8:2 (v/v). Accordingly, these ratios of powder and binder components were optimized by recording voltammograms of solution containing 1.0 mM [Fe(CN)_6_]^3−, 4−^ at the surface of electrodes with different ratios of the components. Also, the stability of ZIF-8 as aqueous solution helps for a repeatable electrochemical response in electroanalytical systems.

### Preparation of DNA/BMIMS/ZIF-8/CPE

DNA/BMIMS/ZIF-8/CPE was perpetrated by the addition of 10 μL of ds-DNA solution (50 mg/L) prepared into acetate buffer (0.5 M, pH 4.8) using a dropwise strategy. Notably, this value was optimized by recording ds-DNA at the surface of DNA/BMIMS/ZIF-8/CPE in the concentration ranged between 10 and 60 mg/L.

### Intercalation Investigation

To study the intercalation of mitoxantrone hydrochloride with ds-DNA at the surface of DNA/BMIMS/ZIF-8/CPE, the electrode was immersed into a solution containing Tris-HCL buffer solution (pH = 7.4) with mitoxantrone hydrochloride and then remained for 12 min under the stringing condition. Afterward, the electrode was washed with the acetate buffer solution and the differential pulse voltammograms of electrode was then recorded in acetate solution (0.5 M, pH 4.8).

### Molecular Docking Study

**Figure 1:**
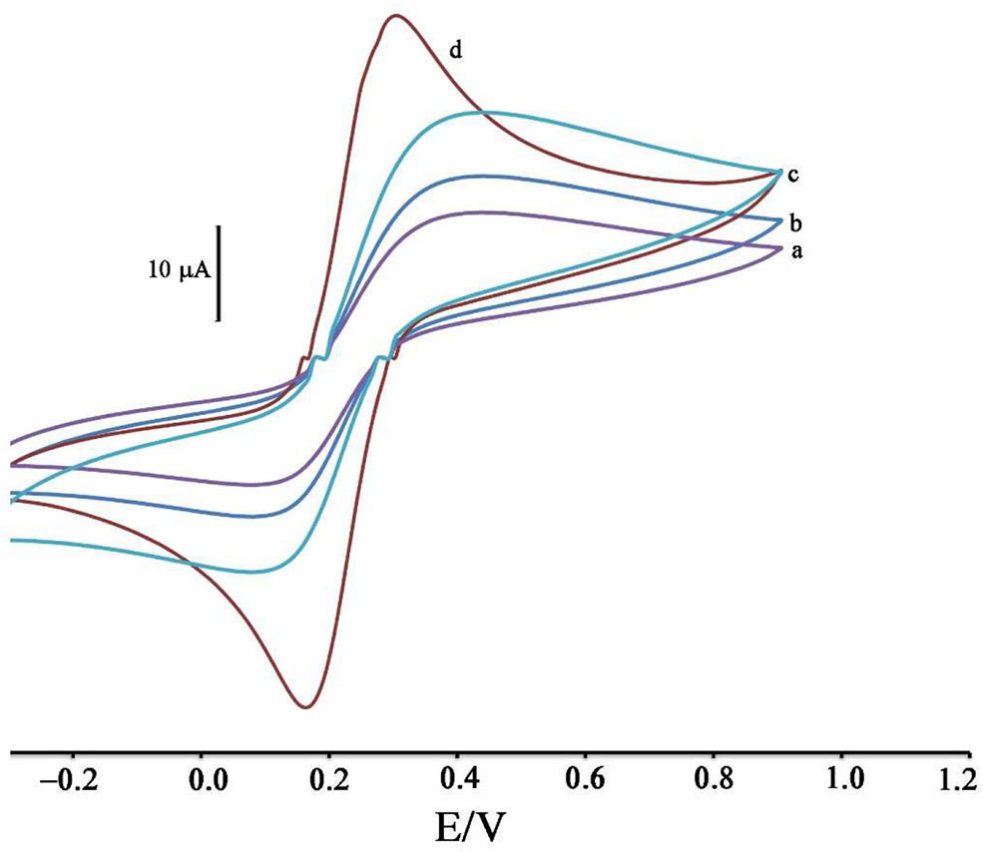
Cyclic voltammograms of solution 1.0 mM [Fe(CN)_6_]^3−/4−^ in the presence of KCl 1.0 M at surface of (a) CPE, (b) ZIF-8/CPE, (c) BMIMS/CPE and (d) BMIMS/ZIF-8/CPE.

In this work, the molecular docking study is performed to evaluate the affinity of mitoxantrone drug in the active site of DNA hexamer d(CGATCG)2 containing an intercalation gap (PDB ID:1Z3F). For a comprehensive investigation of the binding orientation analysis, the best conformer with the lowest root mean square deviation (RMSD) value of 0 Å and the highest binding energy value is selected. The docking of mitoxantrone into the DNA sequence suggests the intercalation of the aromatic rings of mitoxantrone drug between cytosine and guanine base pairs of DNA with a binding energy of −6.7 kcal/mol as shown in Figure 1, curve a. The docked model reveals that the hydrogen and oxygen atoms of mitoxantrone drug have participated as the donor and acceptor to form four intermolecular hydrogen bonds (HBs) with base pairs of DNA (see Figure 1, curve b). It is found that the interaction of oxygen (O5) atoms of drug molecule with H22 atom of deoxyguanosine (DG6 of chain B) of DNA leads to O5…H22-N2 conventional HB with distance of 2.6 Å. Also, the H36 atom of drug as the proton donor interacts with O4′ atom of deoxyribose sugar moiety linked to guanine (DG6 of chain B) as a proton acceptor with a distance of 2.3 Å. Furthermore, the hydrogen atom of the hydroxyl terminal group of drug molecule is bonded to the second and forth oxygen atoms of deoxycytosine (DC5 of chain B) of hexamer of DNA, i.e., O2…H60-O6 and O4′…H60-O6 with the O…H distances of 2.4 and 2.2 Å, respectively. The hydrogen bond angles are 109.5°, 110.5°, 134.5°, and 163.6° for O5…H22-N2, O2…H60-O6, O4′…H60-O6 and O4′…H36-N8, respectively. In addition, the intermolecular interactions between carbonyl groups of mitoxantrone drug and oxygen atoms of DNA sequence, i.e., O2 atom of DC5 chain B, O4′ atom of DG6 chain B and O4′ atom of DG2 chain A with the respective O…O bond lengths of 3.3, 3.5 and 3.4 Å are observed. The docking study approves the interaction between mitoxantrone drug and guanine residues of DNA contributes in the formation of the stable mitoxantrone-DNA complex.

### Real Sample Analysis

Mitoxantrone (12.5 mg/12.5 mL) was purchased from a local pharmacy and then used as a real sample with no pretreatment. The standard addition method was used for analyzing the mitoxantrone concentration in the injection sample using DNA/BMIMS/ZIF-8/CPE.

## Results and Discussion

### Modification Process Investigation

The modification of the CPE surface with BMIMS and ZIF-8 was investigated by recording the cyclic voltammograms of the solution containing 1.0 mM [Fe(CN)_6_]^3−, 4−^. By moving CPE (Figure 1, curve a) to ZIF-8/CPE (Figure 1, curve b), a little improvement was obtained in the oxidation signal of [Fe(CN) _6_]^3−, 4−^ redox solution, which can be related to the creation of a high surface area of ZIF-8 at surface of CPE. After the addition of BMIMS and at a surface of BMIMS/CPE (Figure 1, curve c), the oxidation current of CPE was increase from 14.65 to 24.4 μA that is relative to high conductivity of IL.

After modification of CPE with ZIF-8 and BMIMS, a sharp redox signal with an oxidation current 34.8 μA was observed relative to [Fe(CN)_6_]^3−/4−^ that can be associated with the synergic effects of BMIMS and ZIF-8 at surface of CPE. This amplification can be created under a high sensitivity condition to determine mitoxantrone at surface of DNA/BMIMS/ZIF-8/CPE. In addition, active surface area of CPE, ZIF-8/CPE, BMIMS/CPE and BMIMS/ZIF-8/CPE were calculated about 0.121, 0.163, 0.184, and 0.22 cm^2^ by solution containing 1.0 mM [Fe(CN)_6_]^3−/4−^ and results confirmed that mediators could be increased active surface area of CPE.

### Intercalation Investigation of Mitoxantrone at Surface of DNA/BMIMS/ZIF-8/CPE

**Figure 2:**
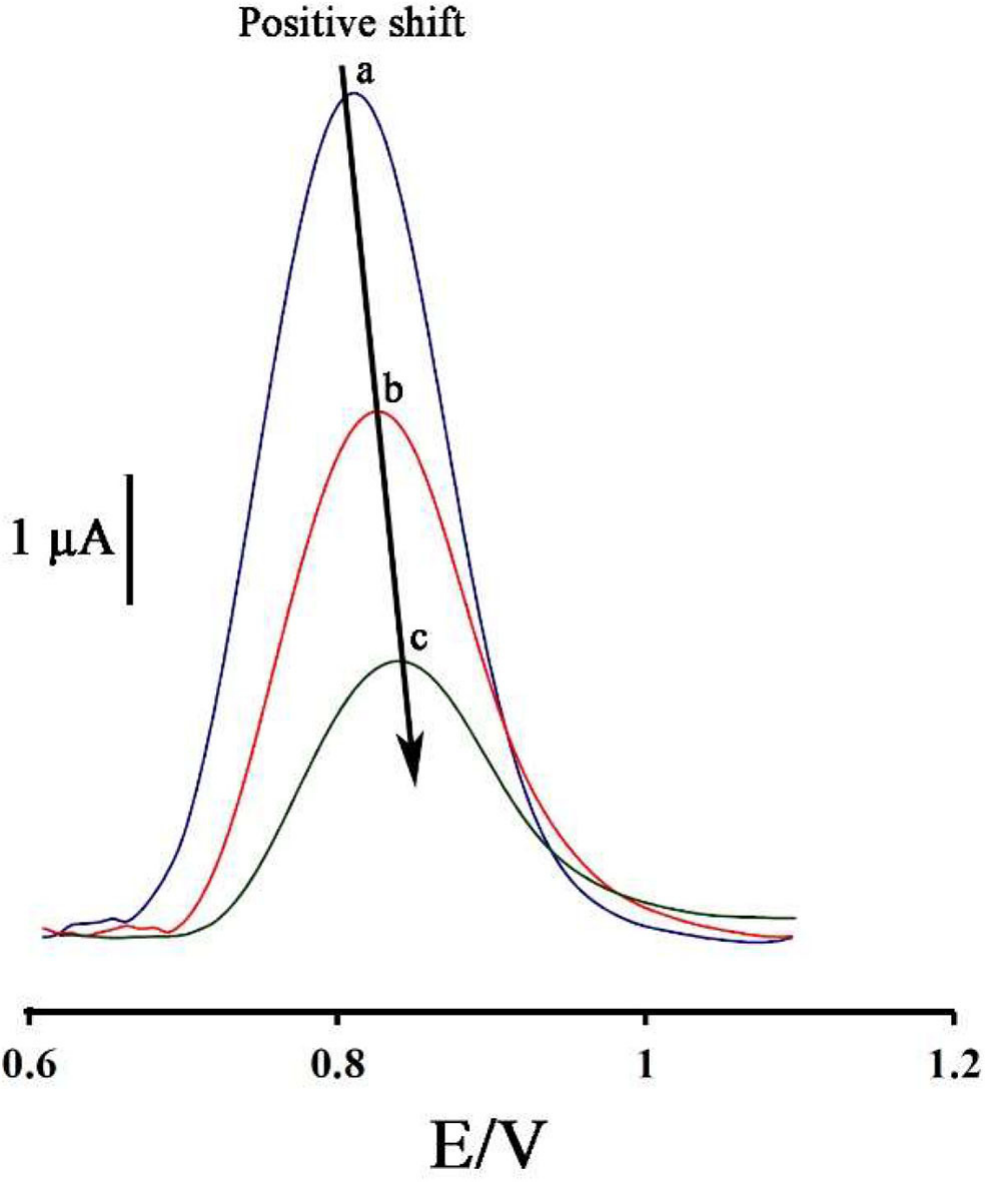
Differential pulse voltammograms of DNA/BMIMS/ZIF-8/CPE in the absence (a) and in the presence of 35.0 μM (b) and 80.0 μM (c) mitoxantrone, respectively.

Figure 2 displays the ds-DNA signal of DNA/BMIMS/ZIF-8/CPE in the absence (curve a) and in the presence of 35.0 and 80.0 μM mitoxantrone (curves b & c), respectively. According to the data reported, the oxidation signal of ds-DNA decreased from 6.2 to 3.87 μA and 2.02 μA in the presence of 35.0 and 80.0 μM mitoxantrone, respectively. Furthermore, the peak potential related to the guanine base shifted from 816 to 825 mV and 845 mV along with the increase of mitoxantrone concentration confirming the intercalation interaction between mitoxantrone and guanine base in ds-DNA structure.

As can be seen, along with increasing of the mitoxantrone concentration, the oxidation current of ds-DNA decreased. Accordingly, this point can be selected as an analytical factor for the determination of mitoxantrone concentration in the solution.

### Optimization of ds-DNA Biosensor for Mitoxantrone Detection

In order to create the best analytical conditions, it is important to optimize the significant factors in the analytical behavior of the biosensor. Therefore, the initial concentration of dsDNA, temperature, buffer types, and incubation time should be optimized.

**Figure 3:**
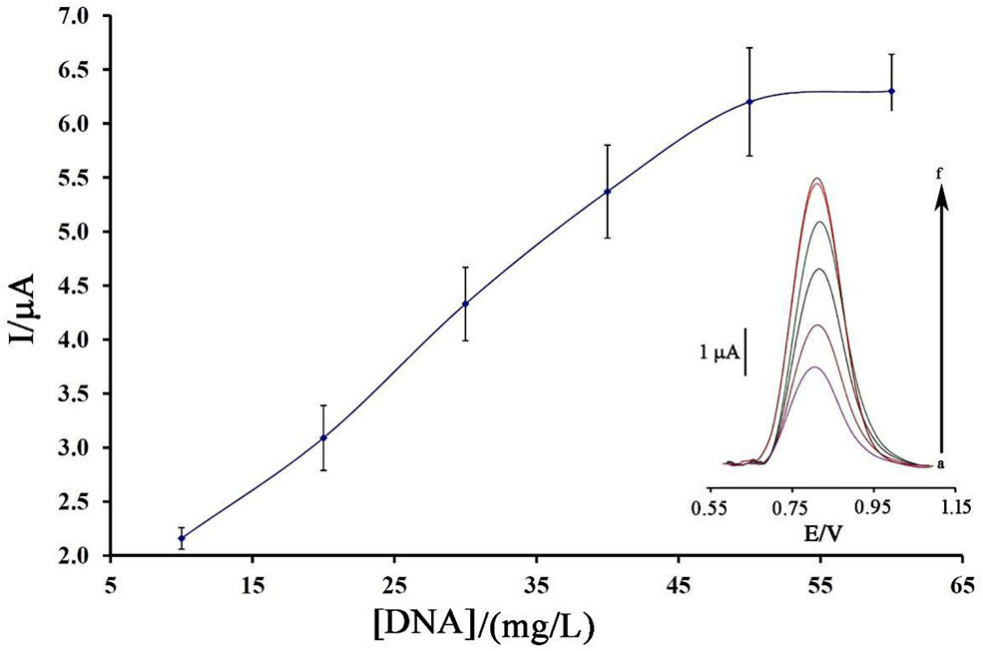
Plot of the oxidation current of ds-DNA compared to its concentration at surface of BMIMS/ZIF-8/CPE (*n* = 4). Inset) Relative differential pulse voltammograms of ds-DNA at surface of BMIMS/ZIF-8/CPE.

Figure 3 displays the oxidation current of ds-DNA compared to the initial concentration of ds-DNA during the modification process.

As can be seen, along with the increase of the initial concentration of ds-DNA, the oxidation signal of DNA/BMIMS/ZIF-8/CPE increased to a concentration of 50 mg/L, which then remained constant. Correspondingly, this point confirms that, in the solution containing 50 mg/L, the electrode surface of BMIMS/ZIF-8/CPE was saturated by ds-DNA, and also the maximum signal can be observed.

**Figure 4:**
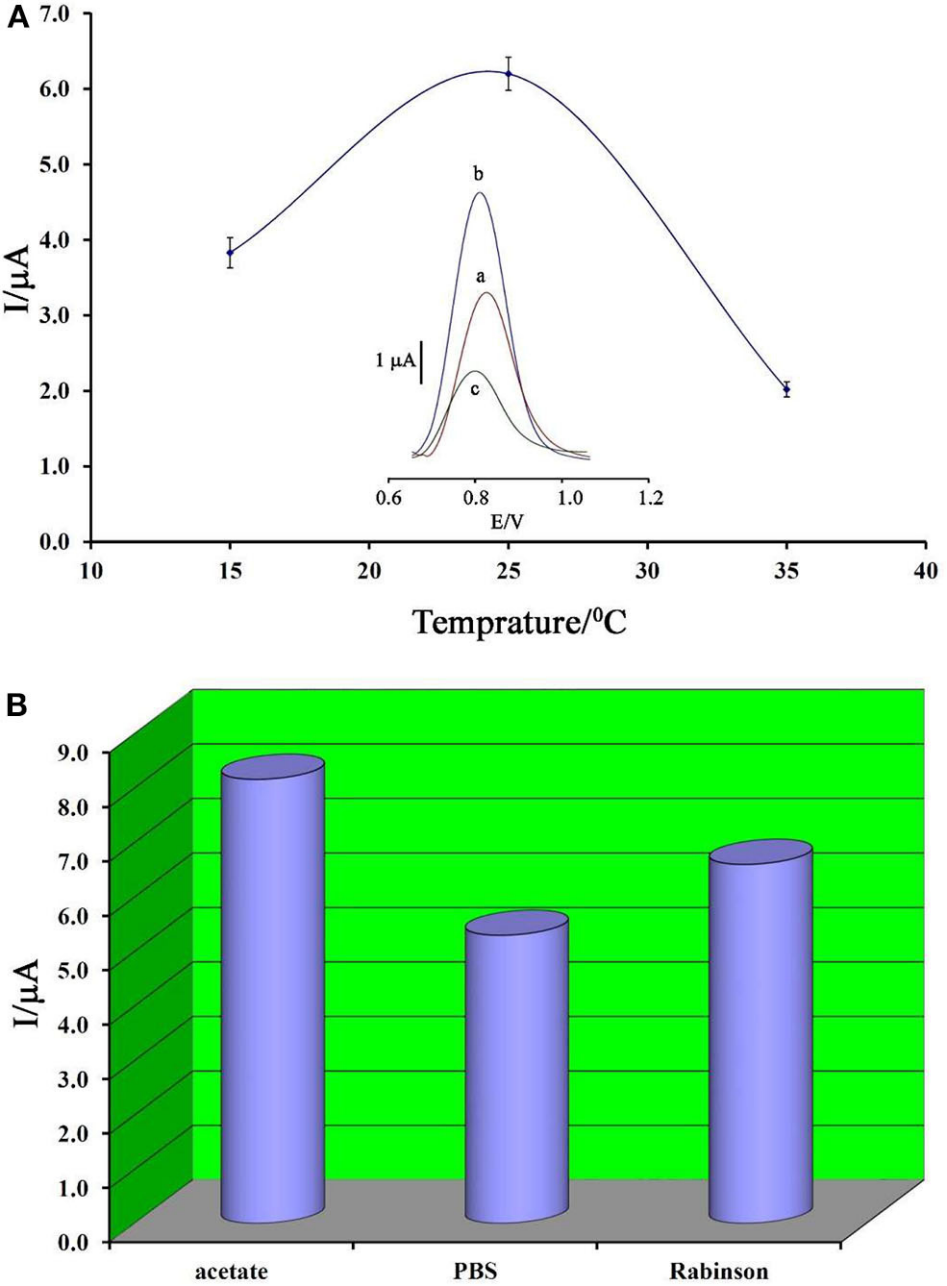
**(A)** Plot of oxidation current of ds-DNA compared to changing in temperature (*n* = 4). Inset) Differential pulse voltammograms of ds-DNA/BMIMS/ZIF-8/CPE recorded at (a) 15°C, (b) 25°C, and (c) 35°C. **(B)** Diagram of oxidation current of ds-DNA compared to the type of buffer recorded under the optimum conditions.

Temperature is known as one of the important factors in the fabrication of a DNA biosensor. In this regard, the changes in the ambient temperature of the test can affect the stability of the DNA at the electrode surface. Therefore, in this research, this factor was optimized. As can be seen in Figure 4A, by increasing the ambient temperature from 15 to 25°C, the ds-DNA signal has increased, and then, along with increasing the temperature up to 35°C, this signal has decreased. This point confirms that, in high temperatures, the electrode surface cannot keep ds-DNA at surface of BMIMS/ZIF-8/CPE.

Accordingly, the decreased viscosity of binders in the carbon paste matrix could be considered as one of the main reasons for this point. In addition, in low temperatures, the activity of ds-DNA was low and DNA/BMIMS/ZIF-8/CPE showed a low oxidation signal.

In addition, the type of buffer is one of the main factors in the deposition of ds-DNA at surface of BMIMS/ZIF-8/CPE. Therefore, the effect of acetate buffer, Britton–Robinson buffer, and phosphate buffer solutions on the deposition step of ds-DNA were investigated. As can be seen in Figure 4B, the best oxidation signal relative to ds-DNA can be detected in the solution containing acetate buffer solution and this buffer was selected as the best condition in the next step of the experiment. Moreover, the interference between the phosphate groups of phosphate buffers or Britton–Robinson buffer can be considered as the most important factor in the creation of a weak ds-DNA signal in these buffers.

**Figure 5:**
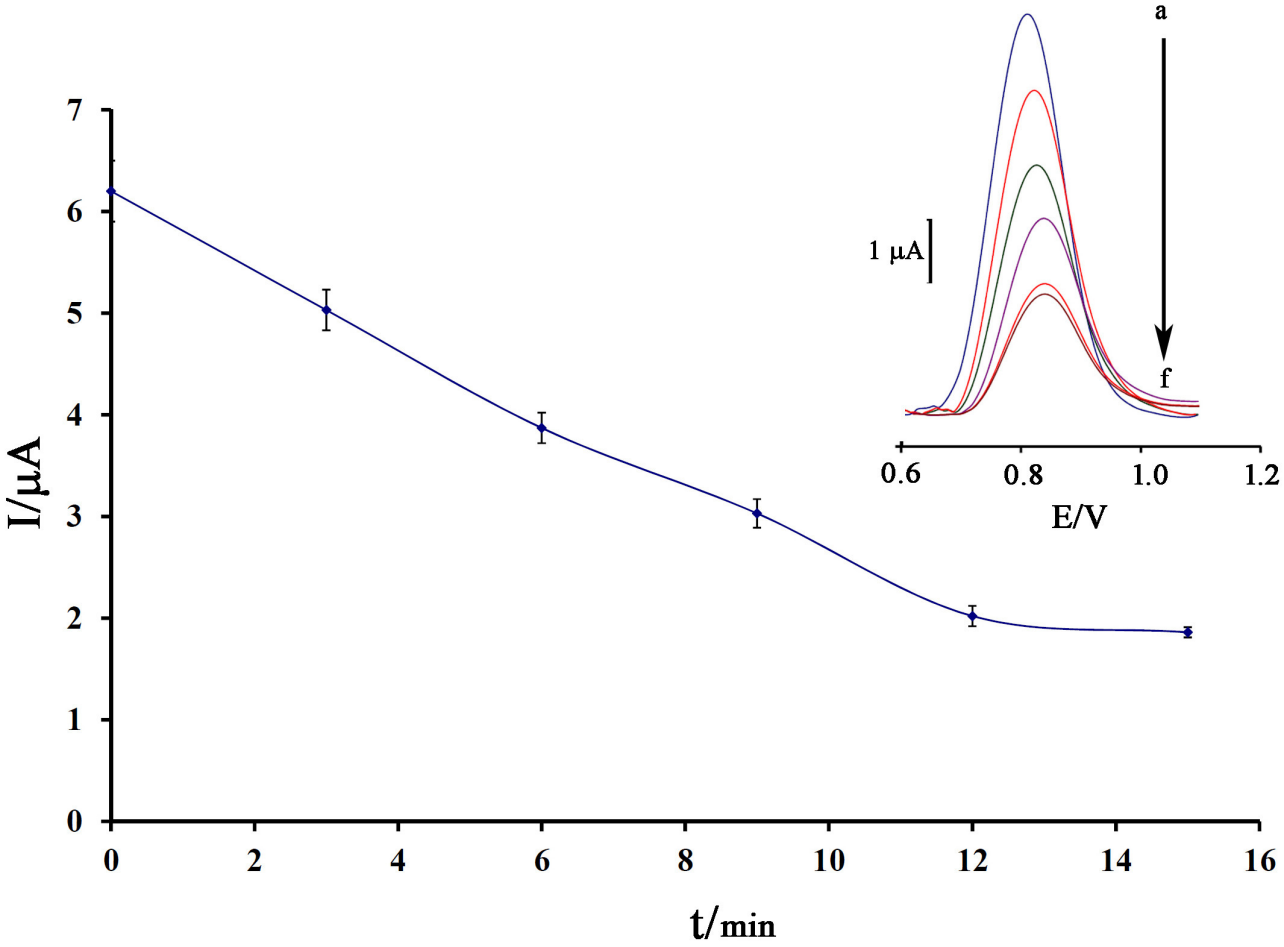
Effect of the incubation time of 80.0 μM mitoxantrone with DNA/BMIMS/ZIF-8/CPE on the guanine oxidation signal (*n* = 4). Inset Relative DPVs in incubation time investigation.

Notably, incubation time is an important factor in the final step of ds-DNA biosensor application in determining anticancer drugs. Also, the low time of the intercalation step does not allow the biosensor to have a proper interaction between the guanine base and the anticancer drugs. Also, in the long term, it is possible to release the ds-DNA-drug into solution and saturation guanine sites in ds-DNA by anticancer drugs. In this regard, the recorded data showed that 12 min is a suitable incubation time for this study (Figure 5).

### Repeatability Sensor Construction

**Figure 6:**
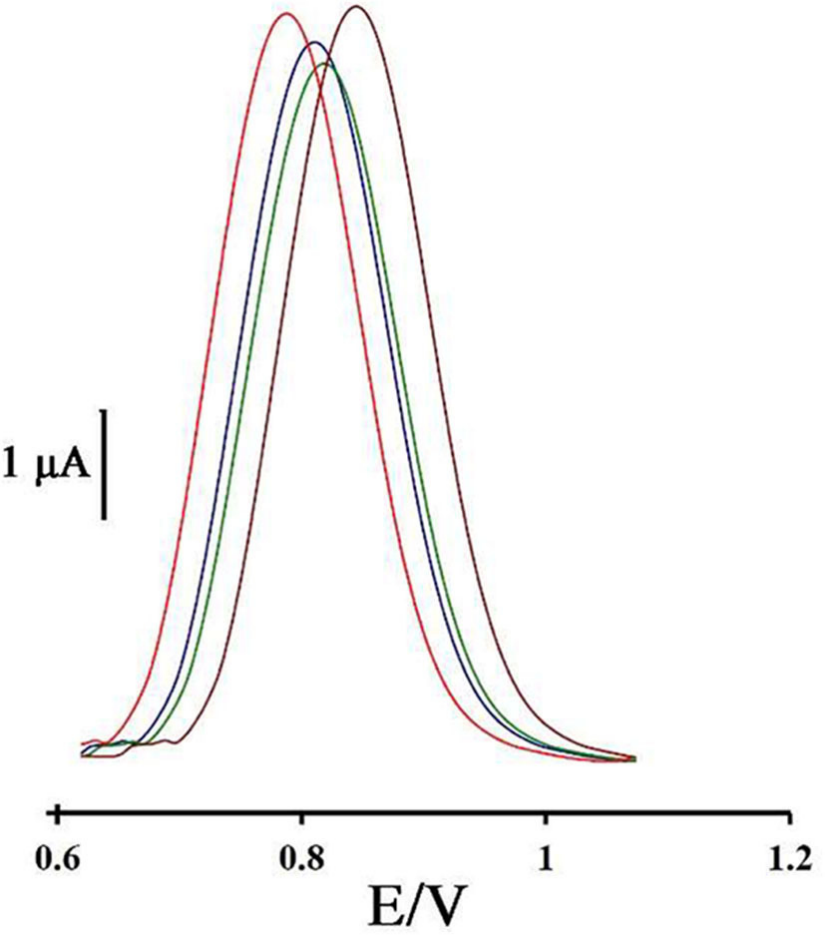
Differential pulse voltammogram of four DNA/ZIF-8/BMIMF/CPE prepared under the same condition.

To investigate the repeatability of the DNA biosensor, four different DNA/BMIMS/ZIF-8/CPE which were fabricated by the same procedure and oxidation signal of guanine were also recorded at the surface of fabricated electrodes (Figure 6). The obtained results showed the relative standard deviation of about 2.7 and 3.1% in the current and potential of guanine signal for four electrodes that are acceptable values for a novel DNA-biosensor.

### Molecular Docking Study

**Figure 7:**
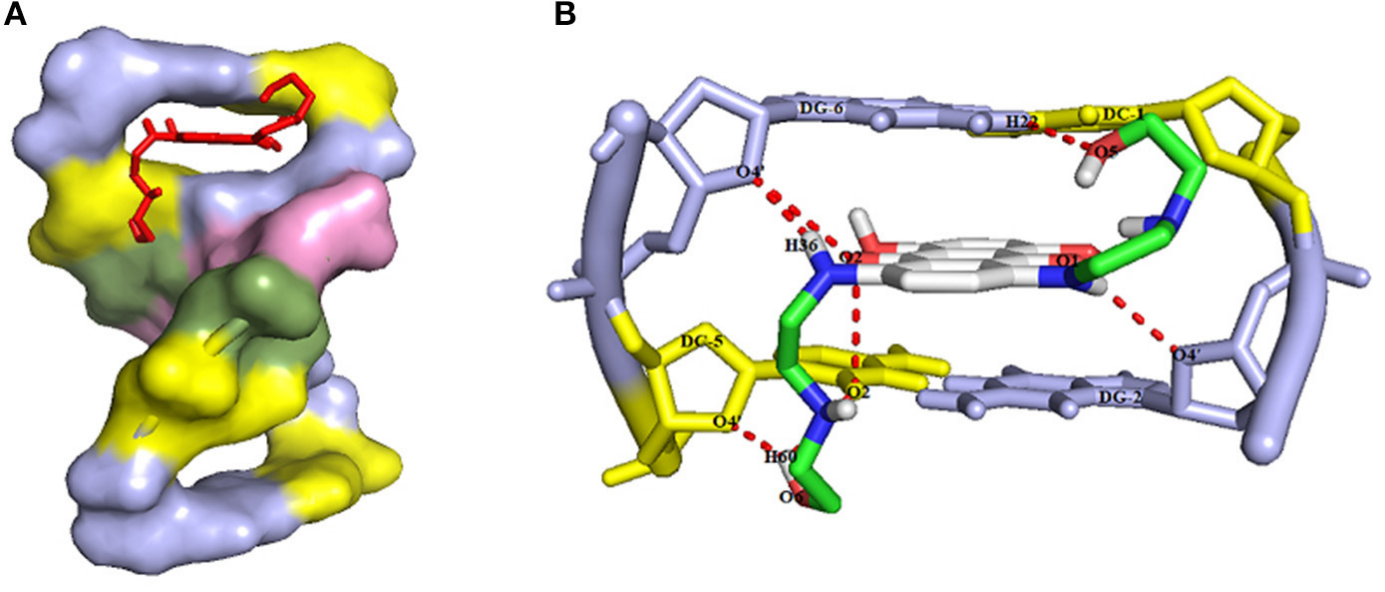
**(A)** The intercalation of MTX drug (red color) into the guanine and cytosine base pairs of DNA receptor (deoxcytosine DC and deoxyguanosine DG in yellow and blue light colors, respectively), and **(B)** The intermolecular hydrogen bond interactions between MTX drug and DNA hexamer d(CGATCG)2 with the numbering atoms discussed earlier in the text.

In this research, the molecular docking study was performed to evaluate the affinity of Mitoxantrone (MTX) drug in the active site of DNA hexamer d (CGATCG) 2 containing an intercalation gap (PDB ID: 1Z3F). For conducting a comprehensive investigation on the binding orientation analysis, the best conformer with the lowest root mean square deviation (RMSD) value of 0 Å and also with the highest binding energy value, was selected. Docking of MTX into DNA sequence suggests the intercalation of the aromatic rings of Mitoxantrone drug between the cytosine and guanine base pairs of DNA with a binding energy of −6.7 kcal/mol, as shown in Figure 7A. The docked model revealed that, the hydrogen and oxygen atoms of MTX drug are participating as the donor and acceptor, to form four intermolecular hydrogen bonds (HBs) with base pairs of DNA, respectively (see Figure 7B). It was found that the interaction of oxygen (O5) atoms of the drug molecule with H22 atoms of deoxyguanosine (DG6 of chain B) of DNA leads to O5…H22-N2 conventional HB with a distance of 2.6 Å. Also, the H36 atom of the drug, as the proton donor, interacts with O4′ atom of deoxyribose sugar moiety linked to guanine (DG6 of chain B), as a proton acceptor with a distance of 2.3 Å. Furthermore, the hydrogen atom of the hydroxyl terminal group of drug molecule is bonded to the second and forth oxygen atoms of deoxycytosine (DC5 of chain B) of hexamer of DNA, i.e., O2…H60-O6 and O4′…H60-O6 with the O…H distances of 2.4 and 2.2 Å, respectively. Moreover, the hydrogen bond angles are 109.5°, 110.5°, 134.5°, and 163.6° for O5…H22-N2, O2…H60-O6, O4′…H60-O6, and O4′…H36-N8, respectively. In addition, the intermolecular interactions between carbonyl groups of MTX drug and oxygen atoms of DNA sequence, such as O2 atom of DC5 chain B, O4′ atom of DG6 chain B, and O4′ atom of DG2 chain A with the respective O…O bond lengths of 3.3, 3.5, and 3.4 Å were observed. The docking study approves the interaction between mitoxantrone drug and guanine residues of DNA that contributes in the formation of the stable MTX-DNA complex.

### Analytical Approach

**Figure 8:**
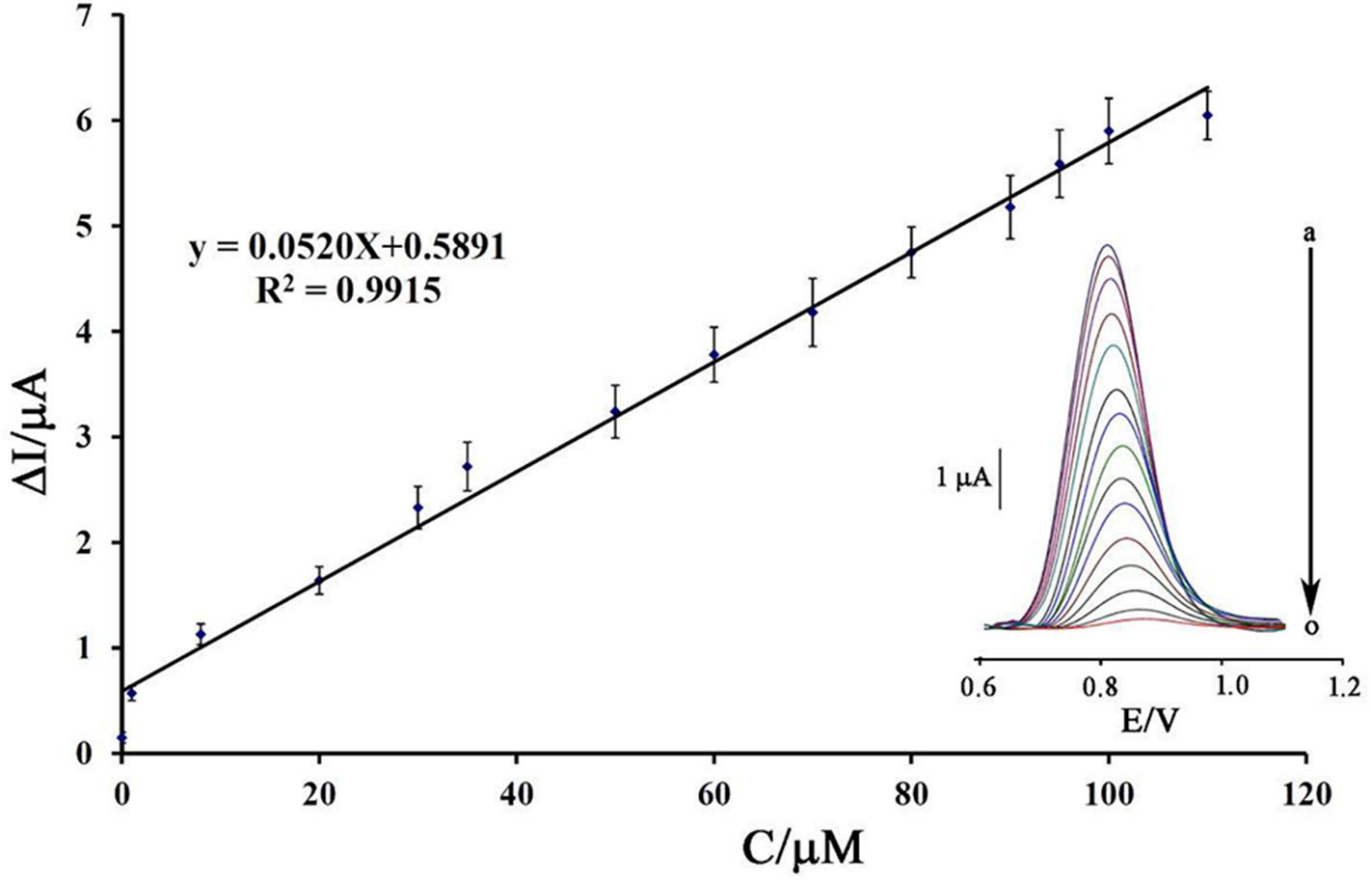
Plot of net current vs. mitoxantrone concentration (*n* = 4). Inset) DP voltammograms of DNA/BMIMS/ZIF-8/CPE in the presence of (a) 0.0; (b) 0.008; (c) 1.0; (d) 8.0; (e) 20.0; (f) 30.0; (g) 35.0; (h) 50.0; (i) 60.0; (j) 70.0; (k) 80.0; (l) 90.0; (m) 95; (n) 100, and (o) 110 μM mitoxantrone.

Using a decreasing trend in DNA signals and its relationship with the concentration of mitoxantrone, a linear dynamic range from 8.0 nM to 110 μM with the equation of ΔI_pa_ = 0.052 C_mitoxantrone_ + 0.589 (*R*^2^ = 0.991) and a detection limit of 3.0 nM was calculated to determine mitoxantrone at surface of DNA/BMIMS/ZIF-8/CPE (Figure 8).

### Kinetic Investigation

Binding energy between guanine and mitoxantrone can be determined by equation 1 as follows:

(1) $$\begin{array}{l} \text{Log}\left[\Delta \text{I}/\left(\Delta {\text{I}}_{\text{max}}-\Delta \text{I}\right)\right]=m\text{ log}\left({\text{K}}_{\text{a}}/\text{M}\right)+m\text{ log}\left(\left[\text{mitoxantrone}\right]/\text{M}\right) \end{array}$$

**Figure 9:**
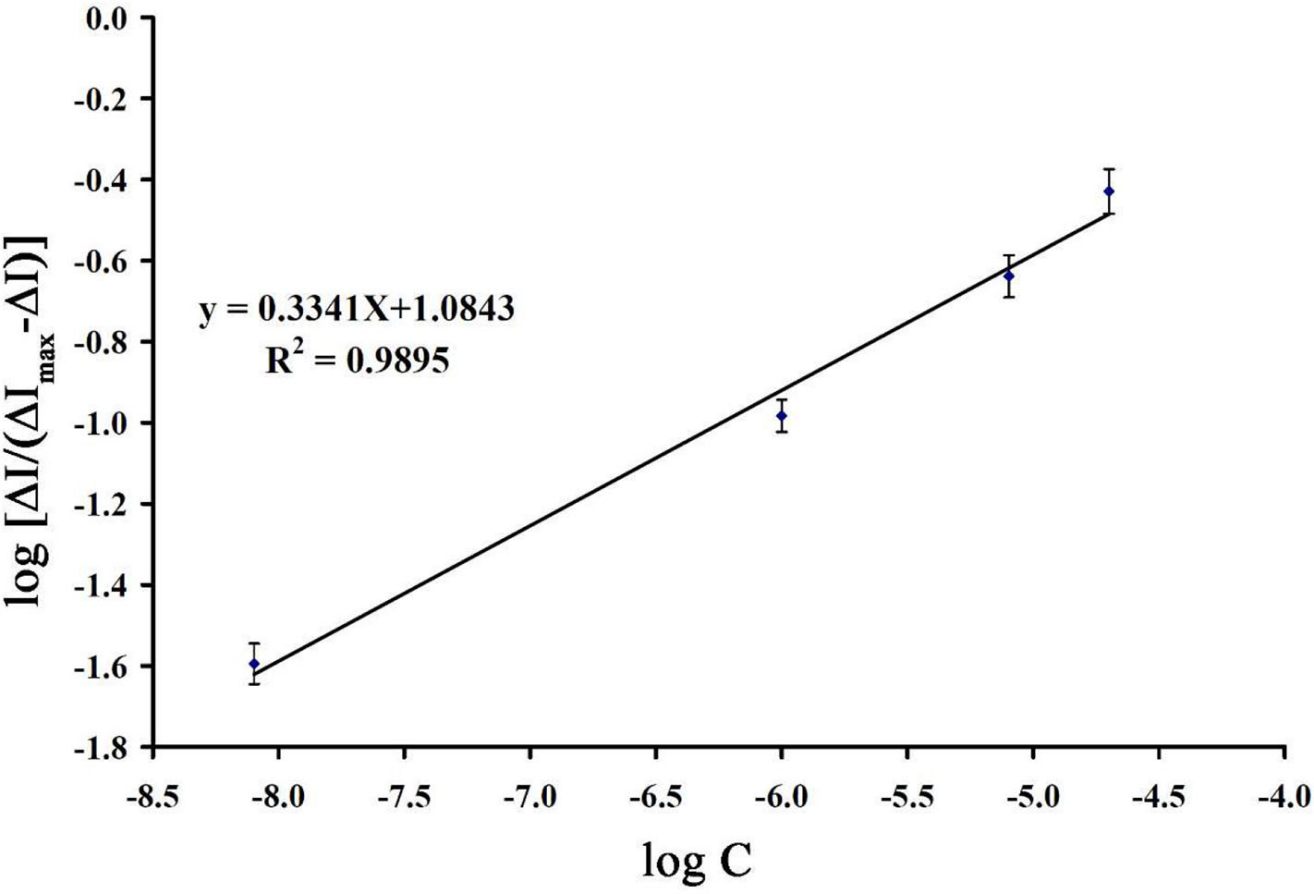
The plot of Log [ΔI/(ΔI_max_ - ΔI)] *vs*. log ([mitoxantrone]/M) (*n* = 4).

Where *m* is the binding number and K_a_ is the association equilibrium constant. Using the slope of recording plot in Figure 9 and equation 1, the values of *m* and K_a_ were determined to be 0.334 and 1.737 × 10^3^ M^−1^, respectively.

### Real Sample Analysis and Selectivity Investigation

The selectivity of DNA/BMIMS/ZIF-8/CPE, as a new biosensor, was investigated in the presence some usual organic and inorganic interference with an acceptable error of 5% in current and potential. The obtained results showed 1,000-fold of ions such as Na^+^, Li^+^, Br^−^, and ${\text{NO}}_{3}^{-}$, 500-fold of methionine, alanine, and phenylalanine, and also 400-fold of vitamin C and vitamin B_2_ had no interference in determination of 20 μM mitoxantrone using the suggested biosensor.

In addition, the ability of DNA/BMIMS/ZIF-8/CPE was checked as a new biosensor for determination of mitoxantrone in the injection samples using the standard addition method. The results are presented in Table 1 and recovery data between 99.18 and 102.08% confirmed the high performance ability of DNA/BMIMS/ZIF-8/CPE for determination of mitoxantrone in real samples.

**Table 1:** The results related to real sample analysis of mitoxantrone by DNA/BMIMS/ZIF-8/CPE (*n* = 4).

**Sample**	**Added (μM)**	**Expected (μM)**	**Founded (μM)**	**Recovery%**
Injection (1)	—	—	1.97 ± 0.21	—
After dilution				
	10.00	11.97	12.22 ± 0.43	102.08
Injection (2)	—	—	2.05 ± 0.28	—
After dilution				
	20.00	22.05	21.87 ± 0.87	99.18

## Conclusion

In this study, a high performance DNA biosensor amplified with ZIF-8 and 1-butyl-3-methylimidazolium methanesulfonate was made-up as a new analytical tool to determine mitoxantrone anticancer drug. The presence of ZIF-8 helps in high loading of ds-DNA and also in improving the quality of the sensor in optimum conditions (T = 25°C; incubation time = 12 min; pH = 4.8 acetate buffer solution and [DNA] = 50 mg/L). In addition, the BMIMS helped as a conductive binder for improving the sensitivity of sensor for trace level analysis of mitoxantrone anticancer drug. Moreover, the DNA/BMIMS/ZIF-8/CPE was successfully used for nano-molar determination of mitoxantrone (LOD = 3.0 nM). In addition, recovery data 99.18–102.08% confirmed the high performance ability of DNA/BMIMS/ZIF-8/CPE as a new biosensor to determine mitoxantrone in the injection samples.

## Data Availability Statement

The original contributions presented in the study are included in the article/supplementary material, further inquiries can be directed to the corresponding author/s.

## Author Contributions

MA: experimental part. PA and SM: characterization of electrochemical results. HK-M: written of paper and electrode modification design. A-MT: Checking the English level of paper and helping in intercalation investigation. All authors contributed to the article and approved the submitted version.

## Conflict of Interest

The authors declare that the research was conducted in the absence of any commercial or financial relationships that could be construed as a potential conflict of interest.
